# Intestinal Mucosal Wound Healing and Barrier Integrity in IBD–Crosstalk and Trafficking of Cellular Players

**DOI:** 10.3389/fmed.2021.643973

**Published:** 2021-03-23

**Authors:** Katrin Sommer, Maximilian Wiendl, Tanja M. Müller, Karin Heidbreder, Caroline Voskens, Markus F. Neurath, Sebastian Zundler

**Affiliations:** ^1^Department of Medicine 1, University Hospital Erlangen, Friedrich-Alexander-Universität Erlangen-Nürnberg, Erlangen, Germany; ^2^Department of Dermatology, University Hospital Erlangen, Friedrich-Alexander-Universität Erlangen-Nürnberg, Erlangen, Germany; ^3^Deutsches Zentrum Immuntherapie (DZI), University Hospital Erlangen, Erlangen, Germany

**Keywords:** wound healing, intestinal epithelial cells, mucosal healing, IBD, intestinal epithelial barrier function

## Abstract

The intestinal epithelial barrier is carrying out two major functions: restricting the entry of potentially harmful substances while on the other hand allowing the selective passage of nutrients. Thus, an intact epithelial barrier is vital to preserve the integrity of the host and to prevent development of disease. Vice versa, an impaired intestinal epithelial barrier function is a hallmark in the development and perpetuation of inflammatory bowel disease (IBD). Besides a multitude of genetic, molecular and cellular alterations predisposing for or driving barrier dysintegrity in IBD, the appearance of intestinal mucosal wounds is a characteristic event of intestinal inflammation apparently inducing breakdown of the intestinal epithelial barrier. Upon injury, the intestinal mucosa undergoes a wound healing process counteracting this breakdown, which is controlled by complex mechanisms such as epithelial restitution, proliferation and differentiation, but also immune cells like macrophages, granulocytes and lymphocytes. Consequently, the repair of mucosal wounds is dependent on a series of events including coordinated trafficking of immune cells to dedicated sites and complex interactions among the cellular players and other mediators involved. Therefore, a better understanding of the crosstalk between epithelial and immune cells as well as cell trafficking during intestinal wound repair is necessary for the development of improved future therapies. In this review, we summarize current concepts on intestinal mucosal wound healing introducing the main cellular mediators and their interplay as well as their trafficking characteristics, before finally discussing the clinical relevance and translational approaches to therapeutically target this process in a clinical setting.

## Introduction

The intestinal mucosa forms a tight barrier with two opposing functions. While it is selectively permeable allowing the absorption of nutrients, it also separates the host from luminal toxins, antigens and microbes that potentially promote disease [reviewed in (1)]. Upon mucosal damage, the epithelial barrier gets leaky facilitating the translocation and therefore excessive exposure of deeper layers of the mucosa to intestinal microbial antigens. This may lead to the recruitment of immune cells releasing different cytokines and may result in disturbed homeostasis [further reviewed in (2, 3)]. Therefore, the regulation of the epithelial barrier function is essential to maintain mucosal homeostasis.

A variety of factors may potentially contribute to mucosal damage, including environmental factors, medication, diet, the host microbiota, infections like HIV as well as genetic factors such as polymorphisms in the CDH1 gene encoding E-Cadherin, which is associated with increased risk to develop ulcerative colitis (UC) [reviewed in (4, 5)]. In general, the pathogenesis of several chronic inflammatory diseases including the inflammatory bowel diseases (IBD) UC and Crohn's disease (CD) is associated with a dysfunctional intestinal epithelial barrier as well as insufficient and delayed mucosal wound healing (6–9). Particularly, wound repair as a pre-requisite to re-establish the mucosal epithelial barrier and intestinal homeostasis is crucial for efficient resolution of inflammation. Hence, mucosal healing (MH) is an increasingly acknowledged goal in IBD therapy in order to achieve and maintain long-term remission. However, mucosal repair and wound healing are complex processes coordinated by the dynamic crosstalk of different cellular players including epithelial cells and infiltrating immune cells as well as their mediators [reviewed in (10)] that are still incompletely understood. A better understanding of these interactions might therefore help to develop tissue-specific approaches to promote wound healing and to treat intestinal inflammation.

In the following paragraphs, we will review the current concepts of intestinal mucosal wound healing, shedding light on the contribution of infiltrating immune cells and their interaction with epithelial cells. Finally, we highlight the clinical relevance of MH and translational approaches to therapeutically target this process.

## Intestinal Epithelial Wound Healing

Intestinal epithelial wound healing is a complex process modulated by various regulatory peptides, including growth factors (GF), and cytokines. Three different phases can be distinguished: Restitution, proliferation, and differentiation and maturation. However, *in vivo*, these processes merge into each other and overlap [reviewed in (11)].

First, epithelial cells surrounding the wound migrate rapidly into the denuded area, form pseudopodia-like structures, re-organize themselves in order to extend into the wound and then re-differentiate after closing the wound defect. This process is termed epithelial restitution and occurs within minutes to hours [reviewed in (12)]. Interestingly, restitution is independent of cell proliferation and one of the most important stimulators of intestinal epithelial cell (IEC) restitution is transforming growth factor β (TGF-β) (13–15). Within the intestinal mucosa, TGF-β is produced by different cell types including epithelial cells, stromal cells, regulatory T cells (T_regs)_, dendritic cells (DC) and macrophages [reviewed in (16)]. Once TGF-β is activated, it enhances restitution by upregulating the expression of matrix metalloproteinase-1 (MMP-1), MMP-10 and a set of genes, including Slc28a2, Tubb2a, and Cpe that are preferentially expressed in fetal IECs (17, 18). Furthermore, mediators, such as vascular endothelial growth factor (VEGF), which are released from the inflamed mucosa, are involved in epithelial cell migration in a TGF-β-dependent manner (19). In addition, it was shown that amino acids like histidine and arginine play an important role in TGF-β-mediated IEC restitution probably via interaction with Smad signaling (20). Furthermore, Lopetuso et al. (21) showed that during acute resolving colitis, IL-33/ST2 promote epithelial repair and restitution by inducing miR-320. It had earlier been demonstrated that miR-320 is decreased in the context of intestinal inflammation, suggesting that this might lead to an inherent defect of epithelial repair (22). Recently, Desmocollin-2 (Dsc2), a desmosomal cadherin exclusively expressed on IECs, was identified as a further key contributor to IEC migration and restitution *in vivo* (23).

In order to increase the number of cells able to resurface the wound area, proliferation is necessary and occurs within hours or days [reviewed in (12)]. This phase is pre-dominantly promoted by various GFs, such as epidermal growth factor (EGF), keratinocyte growth factor (KGF), and fibroblast growth factor (FGF) (24–27), as well as different cytokines including IL-28, which was shown to control proliferation of IECs by activating STAT1 (28), and IL-22, which induces STAT3 signaling, an important regulator of immune homeostasis and mucosal wound healing in the gut (29). Moreover, TLR2 was shown to suppress apoptosis of IECs *in vivo* by selectively regulating trefoil factor 3 (TFF) expression and controlling intestinal epithelial wound repair by modulating epithelial connexin-43 (30, 31).

Finally, differentiation and maturation is needed to re-establish and maintain the mucosal barrier function. Under normal conditions, Lgr5^+^ intestinal stem cells (ISCs), which are located at the base of the crypts, differentiate into short-lived proliferating transit-amplifying progenitors, which further differentiate into absorptive (enterocyte) and secretory progenitors under the control of Wnt/Notch signaling [reviewed in (32, 33)]. Secretory precursors then develop into enteroendocrine cells in a Neurog3-dependent manner or into Goblet or Paneth cells following activation of Atoh1 also known as Math1. Later on, the different cell types acquire their lineage-specific expression of transcription factors (TFs), such as Sox9 for Paneth cells and Klf4 for Goblet cells (34–36). It is also worth mentioning, that there are two distinct ISC populations: Crypt base columnar (CBC) cells, which are actively proliferating and reserve intestinal stem cells (rISC) that are quiescent stem cells until activated upon injury. In line with this, Gonzalez et al. (37) showed that Hopx^+^ cells (rISC) are resistant to injury and are the likely source of epithelial renewal following prolonged ischemic injury (37).

Furthermore, host-microbiota interactions may substantially affect proliferation of epithelial cells and are implicated in intestinal barrier function. E.g., short chain fatty acids (SCFAs) produced by commensal bacteria promote proliferation and differentiation of cells along the crypt-villus axis and, thus, contribute to epithelial restitution (38). Moreover, they are also directly implicated in upholding epithelial integrity to counteract tissue damage (39). In addition to these direct effects on epithelial cells, SCFAs also profoundly impact on the differentiation of mucosal T cells and induce T_regs_ (40), which are involved in mucosal wound healing as described below. Further details on this emerging field are reviewed elsewhere [reviewed in (41, 42)].

Another important cellular mechanism that should be considered in the context of intestinal epithelial wound healing is epithelial-mesenchymal transition (EMT). During this process epithelial cells lose some of their epithelial characteristics, such as polarity and adhesiveness and acquire migratory functions and properties of mesenchymal cells. This transformation is characterized by the interplay of different mediators like TFs, RNAs, and TGF-β family proteins [reviewed in (43)]. In IBD patients, Leeb et al. (44) reported a reduced migratory ability of fibroblasts, which are normally essential in wound contraction during the initial phase of wound healing (44, 45). Based on these findings it is conceivable that epithelial cells are forced to undergo EMT in order to compensate fibroblast dysfunction and to rapidly restore the intestinal barrier function, which, in turn, might predispose for CD-associated fistulae formation (46).

## Contribution of Various Immune Cell Types in Intestinal Repair and Their Interaction With Epithelial Cells

### Lymphocytes and Innate Lymphoid Cells

**Figure 1:**
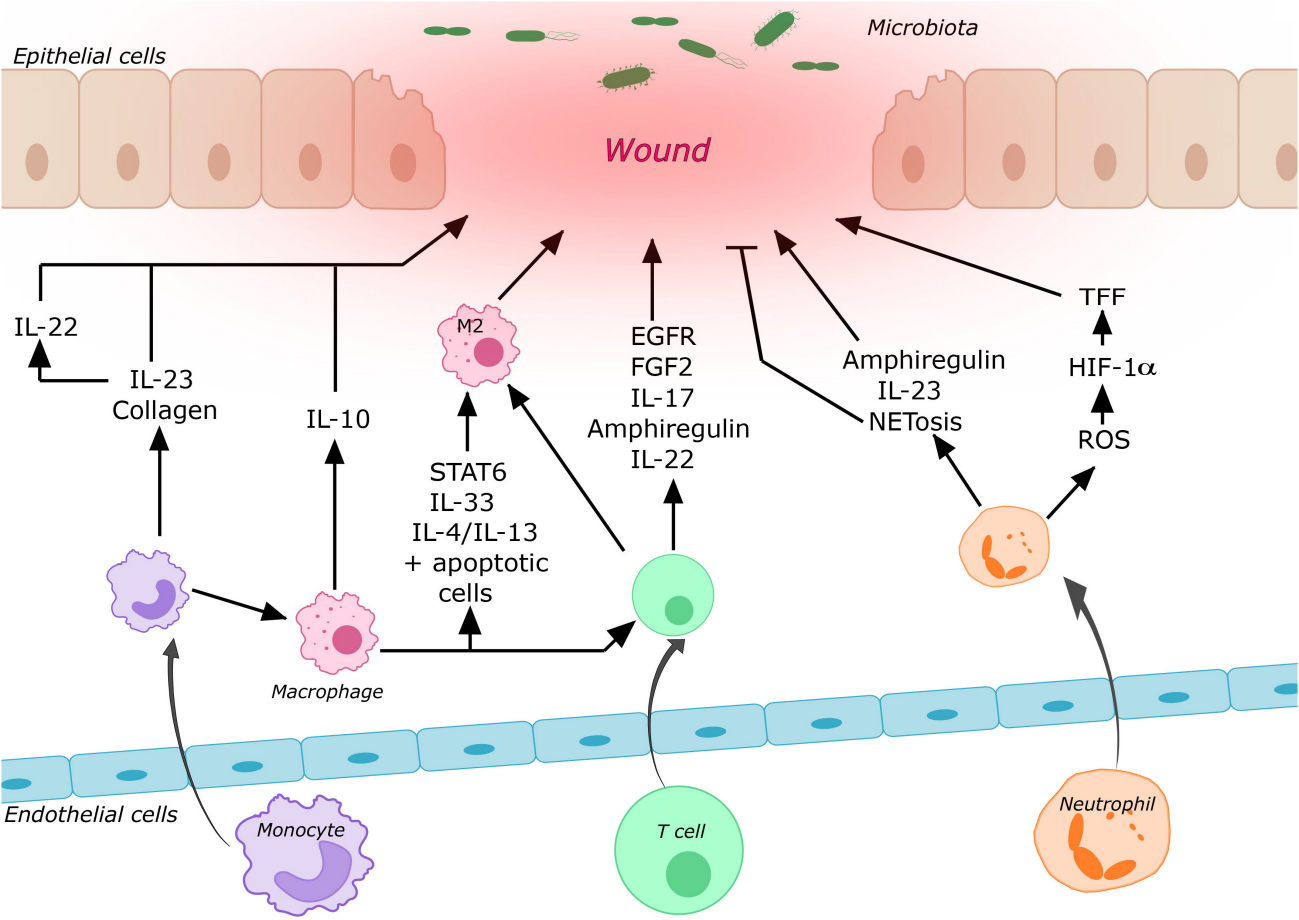
Contribution of some of the most important immune cells to intestinal wound healing. Circulating immune cells are recruited to the wound area by cell trafficking processes. After entering the tissue these cells may undergo differentiation processes and secrete various mediators, which promote or repress mucosal wound healing (for details cf. main text).

Cytokines and other mediators secreted by different T cell subsets play essential roles in wound healing (see Figure 1). Diverse injury models in mice (including models focusing on other organs than the gut, for which evidence is limited) show that depletion of T_regs_ during different phases of wound healing leads to a worse clinical outcome suggesting that they play an important role in the regulation of wound healing probably by counteracting pro-inflammatory stimuli (47–52). Nosbaum et al. (53) showed that T_regs_ in cutaneous wounds attenuated Interferon-γ (IFN-γ) production and reduced the accumulation of pro-inflammatory macrophages. Their elimination resulted in delayed wound re-epithelialization and wound closure. IFN-γ had previously been shown to affect epithelial intercellular junctions and to attenuate intestinal epithelial wound closure by inhibiting epithelial cell migration in a β1 integrin-dependent mechanism (54, 55). Nosbaum et al. (53) were also able to show that, mechanistically, T_regs_ induced the expression of EGFR early after wounding, and lineage-specific deletion of EGFR in T_regs_ resulted in a reduced accumulation and activation as well as increased accumulation of pro-inflammatory macrophages. Furthermore, there is evidence that FGF2 produced by T_regs_ together with IL-17 is involved in gene regulation to repair damaged cutaneous and intestinal epithelium (53, 56). Moreover, CD4^+^CD25^+^Foxp3^+^ T_regs_ isolated from peripheral blood of healthy individuals were reported to induce a phenotypical switch of human monocytes/macrophages to wound healing macrophages (57). Following IL-33 release from damaged epithelia, the GF amphiregulin is another mediator produced by T_regs_, which is involved in limiting inflammation and promoting epithelial repair (47, 58).

Other important cell types involved in intestinal mucosal wound healing are T helper cells (T_H_) and innate lymphoid cells (ILCs). IL-22 is produced by T_H_17 and T_H_22 cells as well as by group 3 ILCs (ILC3) at mucosal surfaces and is a key mediator of this process [reviewed in (59)]. By activating STAT3, IL-22 can not only accelerate proliferation of IECs, but also induce the expression of mucus-associated molecules and the restitution of mucus-producing cells (29, 60). Specifically, IL-22 produced by ILC3s after intestinal injury has been shown to activate intestinal stem cells to promote regeneration (61). Upstream, upon tissue damage, IL-23 may be released leading to the production of IL-22 by ILC3s (62). In line with this, mice deficient for IL-36γ, a potent inducer of IL-23, showed reduced levels of IL-22 and failed to recover from acute intestinal damage. This impaired recovery could be rescued by exogenous IL-23 application (63).

ILC1 show a similar cytokine expression pattern as T_H_1 cells and mainly exhibit their function by secreting tumor necrosis factor α (TNF-α) and IFN-γ to recruit and activate other inflammatory cells (64). As mentioned above, IFN-γ is also involved in the regulation of epithelial barrier integrity (54, 55). Thus, it is not surprising that depletion of intraepithelial ILC1s was associated with reduced proximal colon inflammation in a mouse model of colitis (65).

By contrast, ILC2s produce T_H_2-cell-associated cytokines including IL-4, IL-5, IL-9, and IL-13 [reviewed in (66, 67)]. Upon stimulation by IL-33 and similar to T_regs_, ILC2s produce amphiregulin, which was shown to promote intestinal epithelial cell regeneration in dextran sodium sulfate (DSS)-treated mice (58).

Furthermore, γδ T cells need to be considered when talking about intestinal wound healing as they are the major source of KGF in the mucosa. KGF released from intraepithelial γδ T cells is important for maintaining intestinal epithelial cell proliferation and villus growth, for promoting the repair of epithelial lesions and is also involved in epithelial cell differentiation (68). It was shown that mice lacking γδ T cells have increased susceptibility to DSS-induced colitis and reduced ability to repair damaged epithelia (69). In line with this, Chen et al. (70) found that intraepithelial γδ T cells preserve the integrity of damaged epithelial surfaces by localized delivery of KGF (70, 71).

### Neutrophils

Neutrophils play a crucial role in the first line of defense against microbes. Their antimicrobial machineries include the formation of neutrophil extracellular traps called “NETs” (72) and the elimination of invading microbes through phagocytosis, degranulation and production of reactive oxygen species (ROS) [reviewed in (73)]. These mechanisms are essential for wound healing by on the one hand preventing infection through pathogen translocation, and on the other hand by mediating the early so-called inflammatory phase of wound healing. The recruitment of murine neutrophils to the site of cutaneous injury begins 4 h after the initial injury and peaks after 18 h (74). Depletion of neutrophils in damaged mucosa was shown to lead to a severer colitis as well as impaired recovery and restoration of epithelial integrity (75–77). Furthermore, it was shown that neutrophils enhance the production of amphiregulin by IECs promoting epithelial barrier function and tissue repair (75). Another mechanism contributing to the wound healing properties of neutrophils is their ability to generate a hypoxic microenvironment within the wounded tissue by producing ROS, which in turn leads to the stabilization of HIF-1α in the intestinal mucosa (78). HIF-1α was shown to enhance the epithelial expression of TFF3, which has a barrier-protective function (79). In addition, HIF-1α as a TF promotes the upregulation of genes involved in wound healing including adhesion proteins, different GFs and extracellular matrix components [reviewed in (80)]. Moreover, neutrophils produce IL-22 and IL-23, which are both essential mediators of wound healing as mentioned above (77, 81, 82).

However, neutrophils may also have a negative impact on wound healing. For instance, it was shown that counteracting the alarmin HMGB1 leads to reduced NET formation resulting in improved wound healing and inhibition of NETosis improves wound healing in diabetic mice (83). Furthermore, the accumulation of double strand breaks in the mucosa induced by neutrophils led to impaired wound healing and genomic instability (84). In summary, the effects of neutrophil in this process can be seen as a double-edged sword.

### Monocytes and Macrophages

Circulating monocytes are rapidly recruited to sites of tissue damage or infection, where they further differentiate into inflammatory M1-like macrophages or wound healing M2-like macrophages. Although this classification has been used to explain many experimental observations, it is meanwhile regarded as oversimplification (85).

While the level of CD16 and CD14 expression can be used to differentiate three different monocyte subsets in humans, they are divided into two subpopulations based on their surface expression of Ly6C and/or CX3CR1 in mice (86, 87). Ly6C^hi^ monocytes were shown to be more dominant in the early inflammatory phase exhibiting phagocytic and inflammatory functions, whereas Ly6C^low^ monocytes dominate the later phase displaying anti-inflammatory properties and promoting healing (88). The supportive role of macrophages for barrier function was shown by their ability to increase transepithelial electric resistance and cell height of enteroid monolayers (89). Depletion of macrophages in different mouse models led to severely altered wound morphology, delayed re-epithelialization, reduced collagen deposition, impaired angiogenesis, and decreased cell proliferation in the healing wounds (90, 91). Due to their heterogeneity, macrophages play essential roles in all phases of wound repair. More specifically, depletion after the inflammatory phase increased injury and delayed regeneration while depletion in the early inflammatory phase significantly reduced the formation of vascularized granulation tissue, impaired epithelialization, but also resulted in reduced scar formation in kidneys and skin (92, 93). As mentioned above, IL-23 is an important mediator of wound healing and macrophages were identified as a major source of this cytokine (94). Furthermore, the release of IL-10 by macrophages leads to endothelial cell proliferation and activation of epithelial pro-proliferative pathways in the intestine (95). Interestingly, monocytes and macrophages express virtually all known collagen and collagen-related mRNAs, which is essential for the remodeling phase of wound healing (96). Macrophages also have an impact on other immune cells, e.g., by inducing the differentiation of Foxp3^+^ T_regs_ in the lamina propria (97).

The polarization of macrophages to a wound healing phenotype is essential for repair processes and is regulated by different mediators. Blockade of IL-1β was shown to prime the generation of M2-like macrophages in diabetic mice and IL-33 significantly enhanced intestinal wound healing by promoting the M2 phenotype (98, 99). Moreover, STAT6-mediated M2 polarization promoted repair in 2,4,6-trinitrobenzenesulfonic acid (TNBS) treated mice through activation of the Wnt signaling pathway (100). In addition, IL-4 or IL-13 in combination with apoptotic cells are capable of activating wound healing macrophages. In the absence of apoptotic signals, the proliferation of tissue-resident macrophages, the induction of anti-inflammatory and tissue repair genes are impaired after induction of colitis (101). Recently, Fpr2/3, which is expressed by epithelial cells was shown to regulate the migration of monocytes to sites of mucosal injury, and CX3CR1 was important for the accumulation of macrophages in the wound (102).

However, monocytes and macrophages may also have negative effects on the epithelial barrier. Mononuclear phagocytes interact with IECs by E-Cadherin leading to dysregulated epithelial cell differentiation and intestinal inflammation by disrupting mucosal homeostasis (103, 104). In line with this, a combination of paracrine and hetero-cellular communication between IECs and macrophages was suggested to play a pivotal role in regulating epithelial cell function and dysregulation of intestinal epithelial barrier (105). Sablet et al. demonstrated that inflammatory monocytes contribute to the loss of intestinal barrier function during cryptosporidiosis by producing TNF-α and IL-1β (106).

Taken together, macrophages are crucially involved in many aspects of intestinal wound healing. Depending on their polarization and the phase of wound healing, they may either promote wound closure or predispose for dysregulation of MH.

## Cell Trafficking in the Context of Intestinal Mucosal Wound Healing

As all of the immune cells discussed in the scope of this review are circulating cells or descendants from such cells, there is an obvious need of trafficking for these effectors to reach the site of insult. Thus, cell trafficking should be considered as an integral part of wound healing processes and will shortly be reviewed here.

Described in greater detail elsewhere, cell trafficking describes all processes that are involved in the localization of cells and therefore comprises cellular influx to, retention in and egress from effector tissues [as reviewed in (3, 107)]. Influx from the circulation is regulated by a tightly controlled multistep adhesion cascade. As a prerequisite for transmigration through the endothelium, interaction of selectins and their respective ligands on endothelial cells recruit circulating cells to the vessel walls of high endothelial venules (HEVs) leading to rolling and reduced velocity (108). This slow-down increases the availability of circulating cells to chemotactic stimuli, especially to chemokines, thereby enabling chemokine-induced conformational changes of heterodimeric integrins. Activated integrins are able to firmly bind to endothelial cell adhesion molecules, leading to the arrest of circulating cells on the vessel wall and subsequent para- or intracellular transmigration and target tissue invasion (109).

With regard to gut homing, the α4β7 integrin-mucosal vascular addressin cell adhesion molecule 1 (MAdCAM-1) axis was identified as important due to the virtually exclusive expression of MAdCAM-1 on HEVs of the intestinal tract (110). The relevance of this axis in intestinal wound healing was recently demonstrated, as anti-α4β7 antibody treatment of mice in a colon wound model led to impaired intestinal wound closure, most likely due to reduced homing of non-classical monocytes (NCMs) and a reduction of NCM-derived wound healing macrophages (111). Further, gut specificity in trafficking processes may be provided by the exclusive expression of chemokines in the intestine, as for instance of the CCR9 ligand CCL25 and the GPR15 ligand in the small and large intestine, respectively (112, 113). Their participation in cell recruitment to intestinal wounds has not been studied so far and needs to be further elucidated. Interestingly, both α4β7 and CCR9 are induced on gut-homing T cells through retinoic acid (RA) produced by dendritic cells in the gut-associated lymphoid tissue. With regard to ILCs, it has been shown that this is the case only for ILC1s and ILC3s, while α4β7 expression on ILC2s occurs independent of RA and is already induced in the bone marrow (114). In connection with the above-mentioned roles of ILCs in wound healing, it is tempting to speculate that this might lead to continuous gut homing of amphiregulin-secreting ILC2s promoting homeostasis, while ILC3 recruitment might be regulated by the level of inflammation present. However, it is difficult to envision the consequences for wound healing, since ILC3s not only promote mucosal repair through IL-22, but may also promote inflammation and, thus, secondary tissue injury (115).

Retention of homed cells within the target tissues is either controlled indirectly by the regulation of egress signal receptors or by direct anchoring to tissue structures. A key example of indirect retention is the interaction of CD69 with sphingosine 1-phosophate receptor-1 (S1PR1), leading to degradation of the latter and inhibition of extravasation along the S1P gradient into the bloodstream (116, 117). Further extravasation signals might be provided by the interaction of CCR7 and CCL19 or CCL21, facilitating the recruitment of receptor-bearing cells to the lymphatic system (118, 119). Direct anchoring of recruited cells can be provided by the interaction of integrins with cell adhesion molecules in the tissue. E.g., αE-integrin (CD103) dimerizes with β7-integrin and mediates tissue retention through interaction with E-Cadherin (120, 121). Although the retention of cells in the wound area or their recirculation to the blood will certainly be of relevance in the spatiotemporal orchestration of the wound healing process, since it might lead to the accumulation or reduction of repair-promoting or -impeding cell populations, these mechanisms have not been specifically investigated in this context in the gut. However, and interestingly, there is evidence from skin models, that tissue resident memory T cells (T_RM_ cells), which play important roles in the pathogenesis of IBD (122), promote epithelial wound healing (123, 124).

It is also worth mentioning that mucosal cytokine profiles differ between CD and UC. While UC is dominated by T_H_2-associated cytokines like IL-5, IL-13, IL-9, and IL-4 (125–129), CD is marked by cytokines, such as IFN-γ and IL-2, associated with a T_H_1 phenotype (125, 130). T_H_17 cells and cytokines seem to be involved in both entities (131). At the same time, macroscopic differences in ulcerations between CD and UC exist (132) and inflammation patterns between CD and UC differ with immune cell infiltration restricted to the mucosa of the colon in UC, but being transmural and potentially occurring in the whole gastrointestinal tract in CD [reviewed in (133)]. This strongly suggests that different homing mechanisms apply for immune cells in CD and UC that might also impact wound healing. Interestingly, differences in the expression of gut homing markers on different T cell subsets and differential usage of gut homing pathways in ileal CD as compared to colonic UC have been observed [reviewed in (134, 135)]. However, further dedicated studies are needed to explore this assumption in depth.

Taken together, the implications of immune trafficking for intestinal wound healing are obvious. Particularly, they need to be considered in a therapeutic context, especially when trafficking mechanisms are directly manipulated by antibodies. This also highlights the need for further investigation of the trafficking mechanisms participating in intestinal wound healing.

## Clinical Relevance of Mucosal Healing and Therapeutic Approaches

Mucosal healing (MH), a term coined by Truelove and Witts in 1955 (136), is nowadays considered an important study endpoint and increasingly important treatment goal in IBD. Several clinical trials showed the importance and improved clinical outcomes after achieving MH, defined as absent or low signs of mucosal injury on endoscopy (137–141). In UC and CD, it is associated with long-term remission and reduced need for surgery (142, 143). On tissue level and mechanistically, it is obvious that wound healing and restitution of the intestinal epithelial barrier function are major steps in achieving MH. Consequently, the promotion of wound healing has been suggested as a potential therapeutic tool (144). Calprotectin is a soluble protein in the cytosol of neutrophils and known to be elevated in both the intestinal mucosa and feces of IBD patients (145). Several studies have shown a correlation between low fecal calprotectin (FC) concentration and histological remission as well as MH in UC and CD patients. Therefore, low calprotectin levels might be an early predictor of therapeutic success in terms of MH (146, 147).

One experimental approach to achieve wound healing that was addressed by several studies, but not in the gut, was the promotion of recruitment and polarization of monocytes and wound healing macrophages (148, 149). Maruyama and colleagues (150) showed that upon injection of IL-1β-activated macrophages in mice, the production of VEGF-C was increased and cutaneous wound healing improved. Interestingly, one mechanism of action of corticosteroids is M1 macrophage suppression in response to LPS stimulation, which involves the miR-155 (151). Moreover, neutrophils as cellular mediators can be targeted. In the context of peritonitis, Norling et al. (152) showed that nanoparticles containing aspirin-triggered resolvin D1 or a lipoxin A4 analog reduced polymorphonuclear cell influx and enhanced wound healing. As different GFs like EGF, VEGF, and KGF mediate epithelial repair, they might also be interesting candidates [reviewed in (153)]. Another promising therapeutic approach is targeting IL-22, which is considered to promote epithelial integrity via STAT3. Consequently, an IL-22 IgG4 Fc fusion protein (UTTR1147A) is currently tested in patients with moderate-to-severe UC and CD (ClinicalTrials.gov Identifier: NCT03558152, NCT03650413).

In addition to these experimental concepts, several current IBD treatments were shown to have a protective or regenerative effect on the damaged epithelium and to promote MH [reviewed in (154)]. Aminosalicylates not only affect intestinal inflammation via various signaling pathways such as NF-κB, but also directly stimulate epithelial wound healing by enhancing epithelial cell restitution and proliferation (155–157). Anti-TNF-α antibodies such as infliximab and adalimumab are able to induce and maintain MH (144, 158–160) by restricting the inflammatory infiltrate and T cell proliferation within the lamina propria and by downregulating the expression of metalloproteinases and pro-inflammatory molecules (161). For infliximab, a single nucleotide polymorphism in the TRAP1 gene has been described to be associated with MH in CD patients (162). Moreover, anti-TNF-α antibodies support regenerative processes by reducing inflammation, restoring gut barrier function, mucosal secretion and by activating fibroblasts (163). In addition, it has been suggested that these antibodies mediate Fc region-dependent induction of wound healing macrophages. It was shown that infliximab as well as adalimumab can induce wound healing macrophages *in vitro* and *in vivo* (164, 165). Similarly, ustekinumab, a monoclonal antibody directed against IL-12 and IL-23, successfully induced MH in CD patients (166).

As the JAK/STAT pathway seems to play an important role in the interaction of lymphocytes and IECs through a variety of cytokines, it is not surprising that tofacitinib, a JAK inhibitor routinely used in UC treatment, is able to induce and maintain MH (167). Lechner et al. (168) recently demonstrated that tofacitinib specifically reduces pro-inflammatory cytokines that are produced by lamina propria T cells and affects their homing potential by suppressing the surface integrin expression on T cells. However, in an experimental model of intestinal mucosal wounding, high concentrations of tofacitinib rather prolonged wound healing (168), an observation that requires further translational studies to reconcile it with the clinical outcomes.

Another important class of IBD therapeutics are anti-trafficking agents [reviewed in (3)]. Vedolizumab, a humanized monoclonal anti-α4β7 antibody, inhibits the binding and subsequent migration of lymphocytes into the gut (169). The GEMINI I trial showed that significantly more UC patients treated with vedolizumab than with placebo achieved MH (140, 170). However, mechanistic data explaining the impact of vedolizumab on trafficking of cells implicated in wound healing in inflammation are so far missing. Thus, it is not clear, whether this is a direct effect or secondarily resulting from reduced inflammation and associated changes in the balance of cells promoting and counteracting mucosal repair. In seeming contrast to data on MH as a study endpoint assessing control of inflammation, several (but not all) studies reported that patients treated with vedolizumab are more vulnerable to post-operative complications (171–176). A potential explanation might be that, according to a recent study from our group, blocking α4β7 impaired gut homing of NCMs, which was associated with delayed wound healing and reduced perilesional presence of wound healing macrophages (111). It is important to mention that this is not necessarily contradicting the mentioned MH data, since this study exclusively addressed exogenous tissue injury in the absence of inflammation and it is likely that ongoing inflammation will substantially modulate trafficking, communication and signaling pathways.

Collectively, almost all available therapies for the treatment of IBD have demonstrated their potential to induce MH, although it is not clear to what extent this is a result from direct impact on wound healing processes or a secondary effect of the reduction of inflammation. Thus, further mechanistic data and additional efforts to directly promote wound healing and barrier integrity in the context of IBD are necessary.

## Concluding Remarks

Intestinal mucosal wound repair are key steps for achieving and maintaining MH, which is associated with beneficial clinical outcomes. However, the interplay as well as the trafficking characteristics of the most important cellular mediators like lymphocytes, neutrophils and monocytes/macrophages are not sufficiently characterized. Further research is necessary in order to better understand the contribution of cell trafficking to mucosal wound repair and to base targeted therapeutic approaches on this process.

## Author Contributions

KS, MW, TM, KH, and SZ wrote the manuscript. All authors critically revised the manuscript and approved the final version.

## Conflict of Interest

The authors declare that the research was conducted in the absence of any commercial or financial relationships that could be construed as a potential conflict of interest.
